# Impact of probiotics on muscle mass, muscle strength and lean mass: a systematic review and meta‐analysis of randomized controlled trials

**DOI:** 10.1002/jcsm.13132

**Published:** 2022-11-22

**Authors:** Konstantinos Prokopidis, Panagiotis Giannos, Richard Kirwan, Theocharis Ispoglou, Francesco Galli, Oliver C. Witard, Konstantinos K. Triantafyllidis, Konstantinos S. Kechagias, Jordi Morwani‐Mangnani, Andrea Ticinesi, Masoud Isanejad

**Affiliations:** ^1^ Department of Musculoskeletal Biology, Institute of Life Course and Medical Sciences University of Liverpool Liverpool UK; ^2^ Society of Meta‐research and Biomedical Innovation London UK; ^3^ Department of Life Sciences, Faculty of Natural Sciences Imperial College London London UK; ^4^ School of Biological and Environmental Sciences Liverpool John Moores University Liverpool UK; ^5^ Carnegie School of Sport Leeds Beckett University Leeds UK; ^6^ Department of Pharmaceutical Sciences, Lipidomics and Micronutrient Vitamins Laboratory and Human Anatomy Laboratory University of Perugia Perugia Italy; ^7^ Faculty of Life Sciences and Medicine, Centre for Human and Applied Physiological Sciences King's College London London UK; ^8^ Department of Nutrition & Dietetics Musgrove Park Hospital, Taunton & Somerset NHS Foundation Trust Taunton UK; ^9^ Department of Metabolism, Digestion and Reproduction, Faculty of Medicine Imperial College London London UK; ^10^ Department of Molecular Epidemiology Leiden University Medical Center Leiden Netherlands; ^11^ Department of Medicine and Surgery University of Parma Parma Italy

**Keywords:** Probiotics, Sarcopenia, Muscle mass, Muscle strength, Lactobacillus, Bifidobacterium

## Abstract

Probiotics have shown potential to counteract sarcopenia, although the extent to which they can influence domains of sarcopenia such as muscle mass and strength in humans is unclear. The aim of this systematic review and meta‐analysis was to explore the impact of probiotic supplementation on muscle mass, total lean mass and muscle strength in human adults. A literature search of randomized controlled trials (RCTs) was conducted through PubMed, Scopus, Web of Science and Cochrane Library from inception until June 2022. Eligible RCTs compared the effect of probiotic supplementation versus placebo on muscle and total lean mass and global muscle strength (composite score of all muscle strength outcomes) in adults (>18 years). To evaluate the differences between groups, a meta‐analysis was conducted using the random effects inverse‐variance model by utilizing standardized mean differences. Twenty‐four studies were included in the systematic review and meta‐analysis exploring the effects of probiotics on muscle mass, total lean mass and global muscle strength. Our main analysis (*k* = 10) revealed that muscle mass was improved following probiotics compared with placebo (SMD: 0.42, 95% CI: 0.10–0.74, I^2^ = 57%, *P* = 0.009), although no changes were revealed in relation to total lean mass (*k* = 12; SMD: ‐0.03, 95% CI: −0.19 – 0.13, I^2^ = 0%, *P* = 0.69). Interestingly, a significant increase in global muscle strength was also observed among six RCTs (SMD: 0.69, 95% CI: 0.33–1.06, I^2^ = 64%, *P* = 0.0002). Probiotic supplementation enhances both muscle mass and global muscle strength; however, no beneficial effects were observed in total lean mass. Investigating the physiological mechanisms underpinning different ageing groups and elucidating appropriate probiotic strains for optimal gains in muscle mass and strength are warranted.

## Introduction

Sarcopenia is a progressive skeletal muscle disorder involving the accelerated loss of muscle mass and function that is associated with increased adverse outcomes including falls, functional decline, frailty and mortality in older adults^1^ but can also be observed in younger populations.^2^ The prognosis of sarcopenia is multifactorial and includes physical inactivity,^3^ hormonal imbalances,^4^ sleep disturbance^5^ and malnutrition^6^ that predispose loss of motor neurons and muscle fibres,^7^ overproduction of reactive oxygen species and pro‐inflammatory mediators,^8^ immune senescence^9^ and an overall state of anabolic resistance,^10^ which are pathophysiologically involved in loss of muscle mass and strength.

Nutritional therapies for treating sarcopenia may include achieving a calorie intake of 24–36 kcal/kg body weight/day and a protein intake of 1.0–1.5 g/kg of body mass/day^11^, ^12^ and supplementation with antioxidants,^13^ protein and essential amino acids (EAAs),^14^, ^15^ omega‐3 fatty acids [eicosapentaenoic acid (EPA) and docosahexaenoic acid (DHA)]^16^ and creatine monohydrate.^17^ Emerging evidence has also revealed a prominent role for targeting the gut microbiota to counteract sarcopenia via the gut–muscle axis.^18^ Specifically, probiotics may promote anabolism via greater amino acid absorption^19^, ^20^ and digestion^21^ and down‐regulation of skeletal muscle catabolism by competing with pathogenic gut bacteria involved in the stimulation of pro‐inflammatory pathways through systemic activation of interleukin‐6 (IL‐6) and tumour necrosis factor‐alpha (TNF‐a).^22^

The use of probiotics has shown potential as a nutritional strategy to prevent and/or treat sarcopenia.^23^, ^24^ Several in vivo studies in mice have reported benefits of *Lactobacillus* supplementation on muscle mass preservation and physical function benefits due to a reduction in low‐grade inflammation and increased mitochondrial function,^25^, ^26^, ^27^, ^28^ as well as inhibition of oxidative stress.^29^ Additionally, an increase in muscle strength, but not mass, after gut microbial transplantation from high‐functioning older adults in comparison with transplantation of microbiota from older adults with poor physical performance colonized in mice has been demonstrated.^30^ Recently, a systematic review concluded that probiotic supplementation may not improve performance following resistance and aerobic exercise^31^; however, their findings were primarily focused on markers of cardiorespiratory fitness and did not capture the overall literature around muscle mass and strength. Hence, there is a growing interest in the potential role of probiotics supplementation to improve muscle mass and strength in humans. This systematic review and meta‐analysis of clinical trials aimed to investigate the effect of probiotic supplementation on muscle mass, total lean mass and muscle strength in both young and older adults across the healthspan.

## Methods

This systematic review and meta‐analysis was conducted in accordance with the Preferred Reporting Items for Systematic Reviews and Meta‐Analyses (PRISMA) guidelines.^32^ The protocol was registered in the International Prospective Register of Systematic Reviews (PROSPERO) (CRD: 42022320115).

### Search strategy

Two independent reviewers (K.P. and K.K.T.) searched PubMed, Scopus, Web of Science and Cochrane Library from inception until June 2022. The full search strategy and the search terms used are described in *Table* S1. A manual search of references cited in the selected articles and published reviews was also performed. As a means to minimize bias, grey literature such as published abstracts and dissertations were also screened. The searches were rerun before submission to retrieve any additional studies that met our inclusion criteria. Discrepancies in the literature search process were resolved by a third and fourth investigator (P.G. and K.S.K).

### Inclusion and exclusion criteria

Studies were included based on the following criteria: (i) randomized controlled trials (RCTs); (ii) adults (i.e. >18 years old) irrespective of health status (iii) intervention group received probiotics; and (iv) comparator group receiving nothing (control) or placebo. Published articles were excluded if they (i) were reviews, letters, in vivo or in vitro experiments or commentaries; (ii) were not published as a full text; (iii) included participants younger than 18 years of age; (iv) the intervention included pharmacological agents (e.g. use of anabolic androgenic steroids); and (v) the intervention included enteral nutrition.

### Data extraction and risk of bias

Two authors (K.P. and K.K.T.) extracted data independently, which included name of first author, date of publication, country of origin, number and age of participants, probiotic type (genera and species level), placebo type, dose of probiotic genera/strain, outcome measurements, method of body composition assessment and method of dietary recall assessment. Disagreements between authors were resolved by two independent reviewers (P.G. and K.S.K.). The quality of the included studies was evaluated using the Risk‐of‐Bias 2 (RoB2) tool^33^ and performed by three independent reviewers (K.P., P.G. and K.K.T.). RoB2 is a comprehensive tool used to assess bias in RCTs based on the following domains: (i) randomization process; (ii) deviations from intended interventions; (iii) missing outcome data; (iv) measurement of the outcome; and (v) selection of the reported result.^34^ According to the scoring system, study bias was defined as ‘high’, ‘some concerns’ or ‘low’.

### Endpoints

This meta‐analysis compared changes in muscle mass, total lean mass and muscle strength in participants allocated to a probiotic supplementation or comparator group (placebo or control).

### Statistical analysis

Quantitative data were treated as continuous measurements, and changes in outcomes from baseline to follow‐up were compared between groups to calculate mean differences. For studies presenting multiple indices of muscle strength, pooled means and standard deviations were derived. When units of measurements were inconsistent and could not be converted to units required for inclusion in the analysis, standardized mean differences were applied. In the event that numerical data were not reported, graphical values were calculated using DigitizeIt 2.5 software. Statistical significance was assessed using the random effects model and inverse‐variance method. Any missing standard deviations for changes between baseline and follow‐up among outcome measurements were estimated depending on the availability of either confidence intervals, standard errors and *t* and *P* values or by calculating a correlation coefficient from a known change from baseline standard deviation derived from a similar study.

Statistical heterogeneity of outcome measurements between different studies was assessed using the overlap of their confidence interval (95% CI) and expressed as measurements of Cochran's Q (chi‐square test) and I^2^. The classification of data as moderately heterogeneous was based on I^2^ from 50–74.9% and highly heterogeneous from 75% and above.^35^ Furthermore, sensitivity analyses were performed to evaluate the robustness of reported statistical results by discounting the effect of lifestyle advice (i.e. energy restriction and/or physical activity) on outcome measurements and according to risk of bias of the included studies. Subgroup analyses based on mean participant age, treatment duration, type of probiotic genera, participant health status, method of body composition assessment and country of origin were also performed. Publication bias was assessed using Begg's funnel plots and Egger's linear regression test^36^ using R software. The meta‐analysis was synthesized using Review Manager (RevMan 5.4.1) software.

## Results

**Figure 1:**
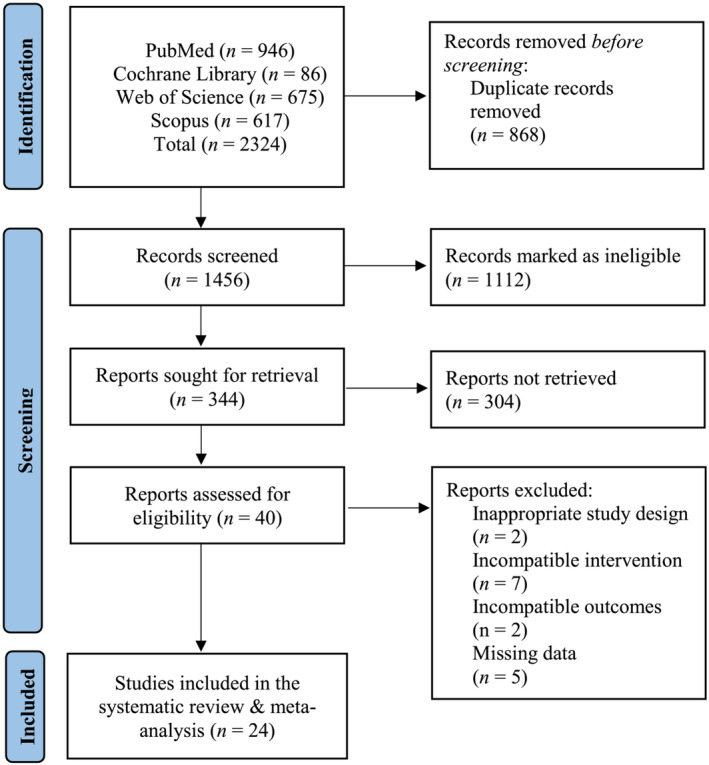
Flowchart of the employed literature search.

The initial literature search provided 2324 publications. Following the exclusion of duplicates and non‐relevant abstracts, 40 full texts were identified as eligible for inclusion in the systematic review and meta‐analysis. Of these 40 studies, seven studies had an incompatible intervention, five studies had missing data, two studies had an inappropriate study design, and two studies had irrelevant outcomes of interest. In total, 24 studies were included in the systematic review and meta‐analysis exploring the effects of probiotics on muscle mass, total lean mass and muscle strength (*Figure* 1). The characteristics of included studies are outlined in *Tables* 1 and 2.

**Table 1:** Study and participant characteristics of the included studies measuring muscle mass and total lean mass

Study	Country	Study design	Health status	Probiotics		Placebo						
				*n* (M/F)	Age	*n* (M/F)	Age	Dose and duration	Intervention	Comparator	Outcomes	Body composition assessment
Chaiyasut 2022	Thailand	Double‐blind RCT	Healthy	24 (3/21)	61.6 (0.8)	24 (7/17)	58.8 (1.2)	2 × 10^10^ ( *Lactobacillus paracasei* HII01, *Bifidobacterium breve*) and 10^10^ ( *Bifidobacterium longum* ) CFU for 12 weeks	*L. paracasei* HII01 *B. breve* *B. longum*	Placebo	Muscle mass (%)	‐
Karim 2022	Pakistan	Double‐blind RCT	Patients with chronic heart failure	44 (44/0)	67.6 (4.9)	48 (48/0)	65.2 (5.6)	11.2 × 10^10^ CFU for 12 weeks	Bifidobacteria ( *B. longum* DSM 24736, *B. breve* DSM 24732, DSM 24737) and Lactobacillus (DSM 24735, DSM 24730, DSM 24733, *Lactobacillus delbrueckii* subsp. Bulgaricus DSM 24734) and *Streptococcus thermophilus* (DSM 24731)	Placebo	Appendicular muscle mass (kg)	BIA
Tarik 2022	India	Double‐blind RCT	Healthy (resistance trained)	28 (28/0)	21.1 (2.5)	28 (28/0)	20.9 (3.1)	2 × 10^9^ CFU for 2 months	*Bacillus coagulans* and 20 g whey protein and 4×/week RT	Placebo and 20 g whey protein and 4×/week RT	TLM (kg)	DXA
Sohn 2022	South Korea	Double‐blind RCT	Overweight	35	47.8 (11.7)	36	45.5 (10.0)	4 × 10^9^ CFU for 12 weeks	*Lactobacillus plantarum* K50 and advice on exercise 3×/week and healthy eating	Placebo and advice on exercise 3×/week and healthy eating	TLM (kg)	DXA
Lee 2021b	Taiwan	Double‐blind crossover RCT	Healthy	8 (8/0)	24.6 (2.8)	8 (8/0)	25.6 (4.1)	20 g for 4 weeks	Synkefir containing *L. paracasei* DSM 32785 (LPC12), *Lactobacillus rhamnosus* DSM 32786 (LRH10), *Lactobacillus helveticus* DSM 32787 (LH43), *Lactobacillus fermentum* DSM 32784 (LF26) and *S. thermophilus* DSM 32788 (ST30) and exercise at 60–80% VO_2_max	Placebo and exercise at 60–80% VO_2_max	Muscle mass (kg)	BIA
Lee 2021c	Taiwan	Double‐blind RCT	Frail	13 (8/5)	80.5 (9.4)	17 (9/8)	75.2 (7.2)	6 × 10^10^ CFU for 18 weeks	*L. plantarum* TWK10	Placebo	Muscle mass (kg)	DXA
Hric 2021	Slovakia	RCT	Healthy	13 (0/13)	51 (12.6)	9 (0/9)	44 (13.0)	30 g for 30 days	Bryndza cheese and weight loss and concurrent training 3×/week	Regular cheese and weight loss and concurrent training 3×/week	Muscle mass (%)	BIA
Hajipoor 2020	Iran	Double‐blind RCT	Obese	28	40.9 (6.8)	31	35.4 (11.7)	4 × 10^7^ CFU each strain for 10 weeks	Probiotic low‐fat yogurt 100 g ( *Lactobacillus acidophilus* La‐B5, *Bifidobacterium lactis* Bb‐12) and low‐calorie diet 500–1000 kcal	Low‐fat yogurt 100 g and low‐calorie diet 500–1000 kcal	TLM (kg)	‐
Lim 2020	South Korea	Double‐blind RCT	Overweight and obese	47	46.4 (12.2)	48	47.2 (11.2)	10 × 10^10^ CFU for 12 weeks	*Lactobacillus sakei* (CJLS03) and exercise ≥3×/week and healthy eating advice	Placebo and exercise ≥3×/week and healthy eating advice	Muscle mass (kg)	DXA
Pan 2020	China	Single‐blind RCT	Metabolic syndrome and type 2 diabetes	15 (7/8)	53.6 (6.8)	16 (8/8)	57.6 (6.1)	Males 90 g Females 75 g for 8 weeks	Fermented noodles ( *L. plantarum* )	Wheat noodles	Muscle mass (kg)	BIA
Huang 2019a	Taiwan	Double‐blind RCT	Healthy	18 (9/9)	Males 22.0 (1.7) Females 23.0 (5.2)	18 (9/9)	Males 22.4 (1.8) Females 20.8 (1.0)	9 × 10^10^ CFU for 6 weeks	*L. plantarum* TW10K	Placebo	Muscle mass (kg)	BIA
Huang 2019b	Taiwan	Double‐blind RCT	Healthy (triathletes)	9	20.2 (0.7)	9	21.1 (1.5)	6 × 10^10^ CFU for 4 weeks	*L. plantarum* PS128 and concurrent training	Placebo and concurrent training	Muscle mass (%)	DXA
Skrypnik 2019	Poland	Double‐blind RCT	Obese	23 (0/23)	56.0 (6.6)	24 (0/24)	60.5 (6.9)	10^10^ CFU for 12 weeks	*Bifidobacterium bifidum* W23, *B. lactis* W51, *B. lactis* W52, *Lactobacillus acidophilus* W37, *Lactobacillus brevis* W63, *Lactobacillus casei* W56, *Lactobacillus salivarius* W24, *Lactococcus lactis* W19 and *L. lactis* W58	Placebo	TLM (%)	BIA
Inoue 2018	Japan	Double‐blind RCT	Healthy	20 (7/13)	69.9 (3.0)	18 (7/11)	70.9 (3.2)	1.25 × 10^10^ CFU each strain for 12 weeks	*B. longum* BB536, *Bifidobacterium infantis* M‐63, *B. breve* M‐16V and *B. breve* B‐3 and RT	Placebo and RT	TLM (kg)	BIA
Nilsson 2018	Sweden	Double‐blind RCT	Low BMD	32 (0/32)	76.4 (1.0)	36 (0/36)	76.3 (1.1)	10^10^ CFU for 12 months	*Lactobacillus reuteri*	Placebo	TLM (kg)	DXA
Minami 2018	Japan	Double‐blind RCT	Overweight	40 (37/3)	45.4 (9.8)	40 (37/3)	45.6 (8.5)	2 × 10^9^ CFU for 12 weeks	*B. breve* B‐3	Placebo	Muscle mass (kg)	BIA
Szulinska 2018	Poland	Double‐blind RCT	Obese	23 (0/23)	55.2 (6.9)	24 (0/24)	58.7 (7.3)	10^10^ CFU for 12 weeks	*B. bifidum* W23, *B. lactis* W51, *B. lactis* W52, *L. acidophilus* W37, *L. brevis* W63, *L. casei* W56, *L. salivarius* W24, *L. lactis* W19 and *L. lactis* W58	Placebo	TLM (kg)	BIA
Toohey 2018	USA	Double‐blind RCT	Healthy (volleyball, soccer athletes)	11 (0/11)	19.6 (1.0) All	12 (0/12)	19.6 (1.0) All	5 × 10^9^ CFU for 10 weeks	*Bacillus subtilis* (DE111) and RT and recovery drink (45 g CHO, 20 g protein, 2 g fat)	Placebo and RT and recovery drink	TLM (lbs)	BIA
Kim 2018	South Korea	Double‐blind RCT	Overweight and obese	26	37.9 (34.7–41.2)	25	38.1 (34.1–42.2)	10^10^ CFU for 12 weeks	*Lactobacillus gasseri* BNR17 and mild energy restriction (200 kcal) and increased physical activity (100 kcal)	Placebo and mild energy restriction (200 kcal) and physical activity (100 kcal)	TLM (kg)	DXA
Osterberg 2015	USA	Double‐blind RCT	Healthy	9 (9/0)	22.4 (1.4)	11 (11/0)	22.9 (0.9)	9 × 10^10^ CFU for 4 weeks	*S. thermophilus* DSM24731, *L. acidophilus* DSM24735, *L. delbrueckii* ssp. Bulgaricus DSM24734, *L. paracasei* DSM24733, *L. plantarum* DSM24730, *B. longum* DSM24736, *B. infantis* DSM24737 and *B. breve* DSM24732 and high‐fat (55% of EI) and hypocaloric diet (+1000 kcal)	Placebo	TLM (kg)	DXA
Minami 2015	Japan	Double‐blind RCT	Overweight	19 (6/13)	58.9 (2.0)	25 (11/14)	61.9 (1.9)	5 × 10^10^ CFU for 12 weeks	*B. breve* B‐3	Placebo	Muscle mass (kg)	BIA
Sharafedtinov 2013	Estonia	Double‐blind RCT	Metabolic syndrome and hypertension	25 (9/16)	52.0 (10.9)	11	51.7 (12.1)	50 g to 1.5 × 10^11^ CFU for 3 weeks	Cheese ( *L. plantarum* TENSIA) and hypocaloric diet (~1500 kcal actual consumption)	Placebo and hypocaloric diet	Muscle mass (kg)	BIA

BIA, bioelectrical impedance; BMD, bone mineral density; CFU, colony‐forming units; DXA, dual‐energy X‐ray absorptiometry; EI, energy intake; RCT, randomized controlled trial; RT, resistance training; TLM, total lean mass;VO2max, maximal oxygen consumption.

Values are presented as mean (± standard deviation).

**Table 2:** Study and participant characteristics of studies measuring muscle strength

Study	Country	Study design	Health status	Probiotics		Placebo					
				*n* (M/F)	Age	*n* (M/F)	Age	Dose and duration	Intervention	Comparator	Indices of physical capacity components
Tarik 2022	India	Double‐blind RCT	Healthy (resistance trained)	28 (28/0)	21.1 (2.5)	28 (28/0)	20.9 (3.1)	2 × 10^9^ CFU for 2 months	*Bacillus coagulans* and 20 g whey protein and 4×/week RT	Placebo and 20 g whey protein and 4×/week RT	Leg press (1RM) Bench press (1RM) Deadlift (1RM)
Karim 2022	Pakistan	Double‐blind RCT	Patients with chronic heart failure	44 (44/0)	67.6 (4.9)	48 (48/0)	65.2 (5.6)	11.2 × 10^10^ CFU for 12 weeks	Bifidobacteria ( *Bifidobacterium longum* DSM 24736, *Bifidobacterium breve* DSM 24732, DSM 24737) and Lactobacillus (DSM 24735, DSM 24730, DSM 24733, *Lactobacillus delbrueckii* subsp. Bulgaricus DSM 24734) and *Streptococcus thermophilus* (DSM 24731)	Placebo	Handgrip strength (kg)
Lee 2021c	Taiwan	Double‐blind RCT	Frail	13 (8/5)	80.5 (9.4)	17 (9/8)	75.2 (7.2)	6 × 10^10^ CFU for 18 weeks	*Lactobacillus plantarum* TWK10	Placebo	Handgrip strength (kg)
Toohey 2018	USA	Double‐blind RCT	Healthy (volleyball, soccer athletes)	11 (0/11)	19.6 (1.0) All	12 (0/12)	19.6 (1.0) All	5 × 10^9^ CFU for 10 weeks	*Bacillus subtilis* (DE111) and RT and recovery drink (45 g CHO, 20 g protein, 2 g fat)	Placebo and RT and recovery drink	Squat (1RM) Deadlift (1RM) Bench press (1RM)
Ibrahim 2018	Malaysia	Double‐blind RCT	Healthy	10 (10/0)	23.0 (1.0)	10 (10/0)	22.0 (2.0)	6 × 10^10^ CFU for 12 weeks	*Lactobacillus acidophilus* , *L. lactis* , *Lactobacillus casei* , *B. longum* , *Bifidobacterium bifidum* and *Bifidobacterium infantis*	Placebo	Knee extension peak torque at 90^o^/s (Nm) Knee flexion peak torque at 90^o^/s (Nm)
Lei 2016	China	Double‐blind RCT	People with non‐displaced distal radius fracture	189 (93/96)	64.3 (4.1)	192 (94/98)	65.1 (3.7)	6 × 10^9^ CFU for 6 months	Semi‐skimmed milk (*L. casei* Shirota)	Semi‐skimmed milk (placebo)	Handgrip strength (kg)

1RM, one‐rep maximum; CFU, colony‐forming units; Nm, newton metre; RCT, randomized controlled trial; RT, resistance training; W, watts.

Values are presented as mean (± standard deviation).

Four studies were conducted in Taiwan,^37^, ^38^, ^39^, ^40^ three in Japan,^41^, ^42^, ^43^ three in South Korea,^44^, ^45^, ^46^ two in China,^47^, ^48^ two in Poland,^49^, ^50^ two in the USA,^51^, ^52^ one in India,^53^ one in Thailand,^54^ one in Malaysia,^55^ one in Iran,^56^ one in Pakistan,^57^ one in Sweden,^58^ one in Slovakia^59^ and one in Estonia.^60^ Twenty‐two studies were double‐blind RCTs,^37^, ^38^, ^39^, ^40^, ^41^, ^42^, ^43^, ^44^, ^45^, ^46^, ^47^, ^49^, ^50^, ^51^, ^52^, ^53^, ^54^, ^55^, ^56^, ^57^, ^58^, ^60^ of which one was crossover double‐blind RCT,^37^ one was a single‐blind RCT^48^ and one was non‐blinded.^59^ Twelve studies provided probiotics in adults aged 50 years and above,^38^, ^41^, ^43^, ^47^, ^48^, ^49^, ^50^, ^54^, ^57^, ^58^, ^59^, ^60^ whereas 12 studies in adults below 50 years of age.^37^, ^39^, ^40^, ^42^, ^44^, ^45^, ^46^, ^51^, ^52^, ^53^, ^55^, ^56^

Eight studies were conducted in adults with overweight and/or obesity,^42^, ^43^, ^44^, ^45^, ^46^, ^49^, ^50^, ^56^ seven in untrained and non‐athletic healthy populations,^37^, ^40^, ^41^, ^51^, ^54^, ^55^, ^59^ one in resistance‐trained individuals,^53^ one in individuals with metabolic syndrome and type 2 diabetes^48^ or hypertension,^60^ one in people with frailty,^38^ one in people with low bone mineral density,^58^ one in patients with chronic heart failure,^57^ one in participants with non‐displaced distal radius fracture,^47^ one in triathletes^39^ and one in volleyball and soccer players.^52^

Eleven studies had a treatment duration of 12 weeks,^41^, ^42^, ^43^, ^44^, ^45^, ^46^, ^49^, ^50^, ^54^, ^55^, ^57^ three of 4 weeks,^37^, ^39^, ^51^ two of 10 weeks,^52^, ^56^ one of 12 months,^58^ one of 6 months,^47^ one of 18 weeks,^38^ one of 2 months,^53^ one of 8 weeks,^48^ one of 1 month,^59^ one of 6 weeks^40^ and one of 3 weeks.^60^

Nine studies assessed muscle mass^37^, ^39^, ^40^, ^42^, ^43^, ^48^, ^54^, ^59^, ^60^ and one study assessed appendicular lean mass,^57^ and 12 studies assessed total lean mass.^38^, ^41^, ^44^, ^45^, ^46^, ^49^, ^50^, ^51^, ^52^, ^53^, ^56^, ^58^ Body composition assessment was performed via bioelectrical impedance (BIA) in 12 studies^37^, ^40^, ^41^, ^42^, ^43^, ^48^, ^49^, ^50^, ^52^, ^57^, ^59^, ^60^ and dual energy X‐ray absorptiometry (DXA) in eight studies,^38^, ^39^, ^44^, ^45^, ^46^, ^51^, ^53^, ^58^ whereas two studies did not report details.^54^, ^56^

Three studies assessed handgrip strength,^38^, ^47^, ^57^ two bench press^52^, ^53^ and deadlift one repetition maximum (1RM) strength,^52^, ^53^ one squat^52^ and leg press 1RM^53^ and one knee extension and flexion peak torque.^55^

Eleven studies utilized products containing *Lactobacillus* species only,^38^, ^39^, ^40^, ^44^, ^45^, ^46^, ^47^, ^48^, ^58^, ^59^, ^60^ five *Bifidobacterium* only,^41^, ^42^, ^43^, ^52^, ^53^ three Lactobacillus combined with *Bifidobacterium*,^54^, ^55^, ^56^ three *Lactobacillus* and *Streptococcus* ^37^, ^51^, ^57^ and two *Lactobacillus*, *Bifidobacterium* and *Lactococcus*.^49^, ^50^

In terms of dose, five studies supplemented probiotic products with a total of 10^10^ colony‐forming units (CFU),^45^, ^46^, ^49^, ^50^, ^58^ three used 6 × 10^10^ CFU,^38^, ^39^, ^55^ three had a fixed dose for each substrain,^41^, ^54^, ^56^ two 9 × 10^10^ CFU,^40^, ^51^ two 2 × 10^9^ CFU,^42^, ^53^ one 11.2 × 10^10^ CFU,^57^ one 5 × 10^10^ CFU,^43^ one 5 × 10^9^ CFU,^52^ one 6 × 10^9^ CFU,^47^ one 4 × 10^9^ CFU,^44^ and one study provided participants with fermented cheese (50 g),^60^ one with fermented noodles (males: 90 g; females: 75 g),^48^ one with Bryndza cheese (30 g)^59^ and one with synkefir (20 g).^37^

### Assessed domains of sarcopenia

Muscle mass and total lean mass were estimated by DXA and BIA. Handgrip strength was expressed in kilograms (kg) and assessed using a hydraulic dynamometer. Leg strength was expressed in kg when assessed via squat, leg press and deadlift 1RM and in newton‐metres when assessed via knee extension and flexion peak torque at 90°/s. Indices of upper body strength were expressed in kg when assessed via bench press 1RM and in Nm when assessed via elbow flexion isometric peak torque. Global muscle strength was expressed as the composite score of all the strength measures using standardized mean difference (SMD).

### Probiotic supplementation and muscle mass

**Figure 2:**
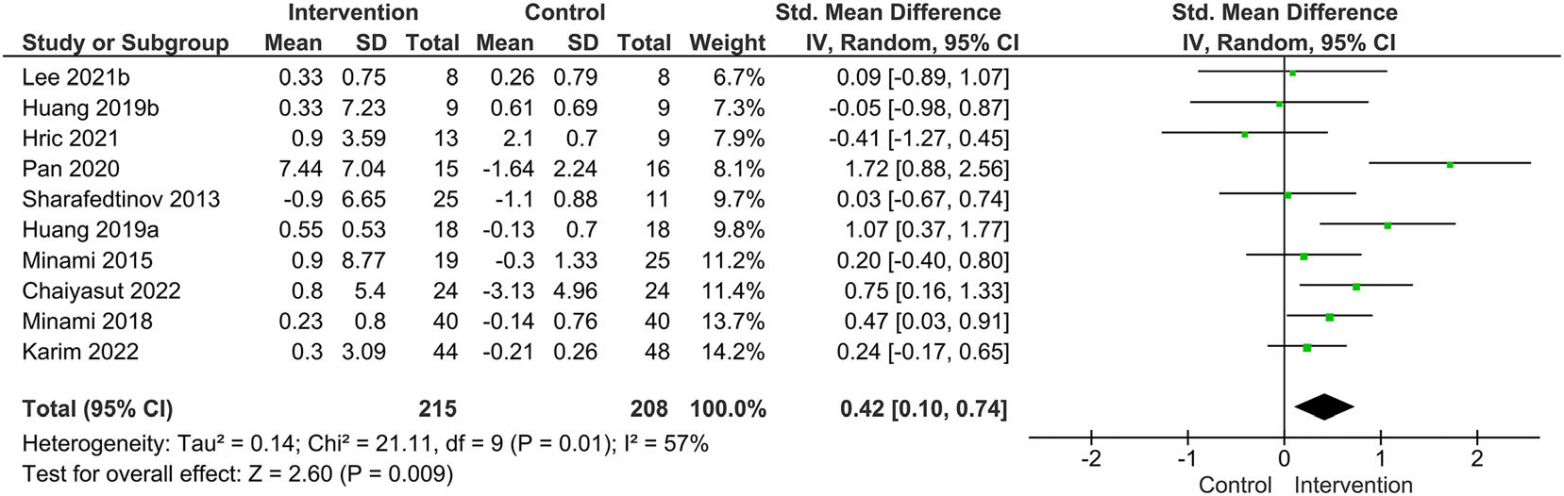
Effect of probiotic supplementation on muscle mass.

Our main analysis (*k* = 10) revealed that muscle mass was improved following probiotic supplementation compared to placebo and displayed a moderate degree of heterogeneity among the included RCTs (SMD: 0.42, 95% CI: 0.10–0.74, I^2^ = 57%, *P* = 0.009) (*Figure* 2).

Based on an array of subgroup analyses, we did not observe an effect of probiotic supplementation on muscle mass in individuals with overweight or obesity (*k* = 5, SMD: 0.46, 95% CI: −0.06–0.97, I^2^ = 66%, *P* = 0.08) and healthy untrained individuals (*k* = 4, SMD: 0.43, 95% CI: −0.20–1.06, I^2^ = 63%, *P* = 0.18) (*Figure* S1) and individuals above 50 years of age (≥50 years ➔ *k* = 6, SMD: 0.41, 95% CI: −0.06–0.88, I^2^ = 69%, *P* = 0.09); however, we found a significant increase in muscle mass following probiotic supplementation in individuals below 50 years of age (<50 years ➔ *k* = 4, SMD: 0.47, 95% CI: 0.03–0.91, I^2^ = 35%, *P* = 0.04) (*Figure* S2). In addition, probiotic supplementation significantly improved muscle mass in the long term (≥12 weeks) (*k* = 4, SMD: 0.39, 95% CI: 0.15–0.63, I^2^ = 0%, *P* = 0.002), but not in the short term (<12 weeks) (*k* = 6, SMD: 0.42. 95% CI: −0.23 – 1.07, I^2^ = 73%, *P* = 0.20) (*Figure* S3). No significant changes were observed after stratification for isolated probiotic species such as *Lactobacillus* (*k* = 7, SMD: 0.48, 95% CI: −0.15–1.01, I^2^ = 69%, *P* = 0.08); however, a significant improvement in muscle mass was found following *Bifidobacterium* (*k* = 2, SMD: 0.37, 95% CI: 0.02–0.73, I^2^ = 0%, *P* = 0.04) (*Figure* S4). Based on geographical location, we observed a significant increase in muscle mass in countries located in Asia (*k* = 7; SMD: 0.61, 95% CI: 0.22–1.01, I^2^ = 56%, *P* = 0.002), but not from countries in Europe (*k* = 2, SMD: −0.15, 95% CI: −0.69–0.40, I^2^ = 0%, *P* = 0.60) (*Figure* S5). Details related to subgroups analyses exploring the impact of probiotic supplementation on muscle mass are shown in *Table* S2. Furthermore, sensitivity analysis based on method of body composition assessment revealed a significant improvement in muscle mass with probiotic supplementation via BIA (*k* = 9, SMD: 0.46, 95% CI: 0.12–0.80, I^2^ = 60%, *P* = 0.007) (*Figure* S6) and based on studies with low to moderate risk of bias (*k* = 10, SMD: 0.46: 95% CI: 0.13–0.80, I^2^ = 60%, *P* = 0.007) (*Figure* S7).

### Probiotic supplementation and total lean mass

**Figure 3:**
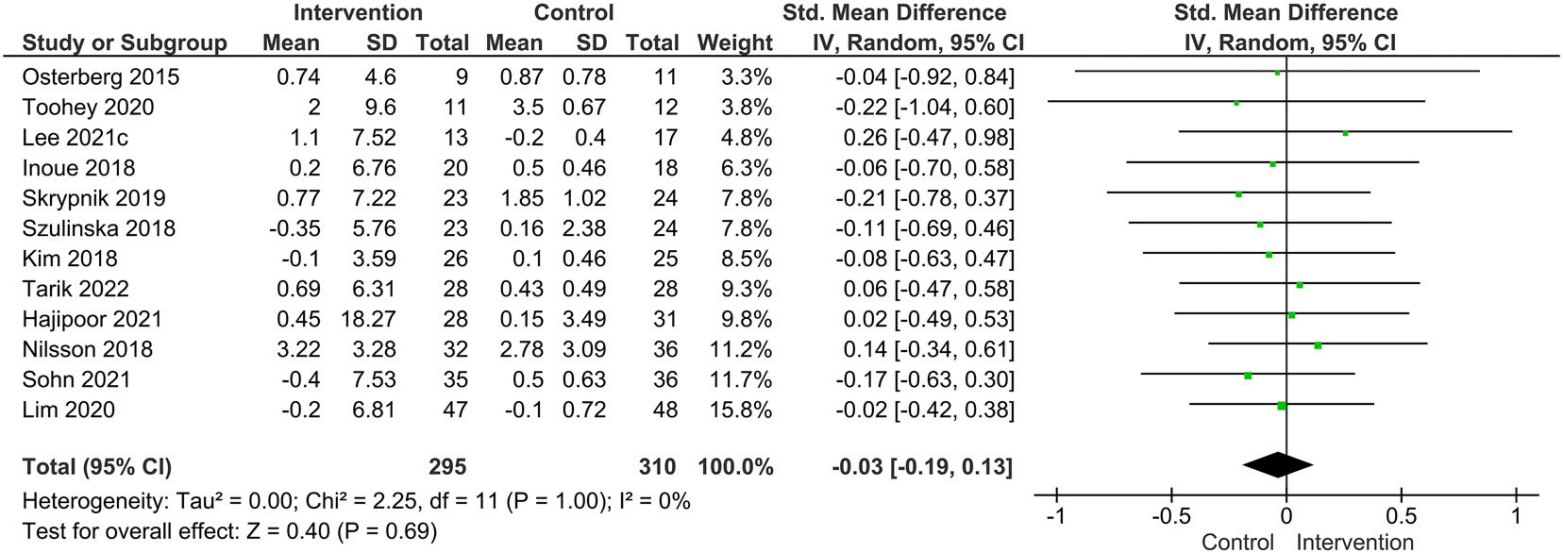
Effect of probiotic supplementation on lean body mass.

Our main analysis (*k* = 12) revealed that total lean mass did not improve after probiotic supplementation compared to placebo and there was a low degree of heterogeneity among trials (SMD: −0.03, 95% CI: −0.19–0.13, I^2^ = 0%, *P* = 0.69) (*Figure* 3).

Based on a series of subgroup analyses, we did not observe any changes in total lean mass following probiotic supplementation in individuals with overweight or obesity (*k* = 8, SMD: −0.03, 95% CI: −0.21–0.15, I^2^ = 0%, *P* = 0.73), healthy untrained (*k* = 2, SMD: −0.05, 95% CI: −0.57–0.46, I^2^ = 0%, *P* = 0.84) or athletic populations (*k* = 2, SMD: −0.02, 95% CI: −0.46–0.42, I^2^ = 0%, *P* = 0.92) (*Figure* S8). In addition, no changes in total lean mass were observed with probiotic supplementation in individuals above and below 50 years of age (≥50 years ➔ *k* = 7, SMD: ‐0.03, 95% CI: −0.25–0.19, I^2^ = 0%, *P* = 0.79), (<50 years ➔ *k* = 5, SMD: −0.03, 95% CI: −0.26–0.19, I^2^ = 0%, *P* = 0.77) (*Figure* S9). Furthermore, probiotics did not improve total lean mass in the short term (<12 weeks) (*k* = 4, SMD: −0.01, 95% CI: −0.32–0.30, I^2^ = 0%, *P* = 0.96) and the long term (≥ 12 weeks) (*k* = 8, SMD: −0.04, 95% CI: −0.23–0.14, I^2^ = 0%, *P* = 0.66) (*Figure* S10) or after categorization for isolated probiotic species such as *Lactobacillus* (*k* = 7, SMD: −0.00, 95% CI: −0.20–0.20, I^2^ = 0%, *P* = 0.99), *Bifidobacterium* (*k* = 3, SMD: −0.03, 95% CI: −0.40–0.33, I^2^ = 0%, *P* = 0.85) or a combination of *Lactobacillus*, *Bifidobacterium* and *Lactococcus* (*k* = 2, SMD: −0.16, 95% CI: −0.57–0.24, I^2^ = 0%, *P* = 0.44) (*Figure* S11). Moreover, based on geographical location, no changes were observed in countries in Asia (*k* = 6, SMD: −0.03, 95% CI: −0.24–0.19, I^2^ = 0%, *P* = 0.80), Europe (*k* = 3. SMD: −0.04, 95% CI: −0.35–0.27, I^2^ = 0%, *P* = 0.82) (*Figure* S12). Subgroup analysis based on method of body composition assessment revealed no changes after evaluation with either BIA (*k* = 4, SMD: −0.14, 95% CI: −0.46–0.17, I^2^ = 0%, *P* = 0.37) or DXA (*k* = 8, SMD: 0.01, 95% CI: −0.18–0.19, I^2^ = 0%, *P* = 0.95) (*Figure* S13). Details related to subgroups analyses exploring the impact of probiotic supplementation on total lean mass are shown in *Table* S3. Finally, sensitivity analysis based on studies with low to moderate risk of bias revealed findings consistent with our initial analysis (*k* = 11, SMD: −0.03: 95% CI: −0.19–0.14, I^2^ = 0%, *P* = 0.76) (*Figure* S14).

### Probiotic supplementation and global muscle strength

**Figure 4:**
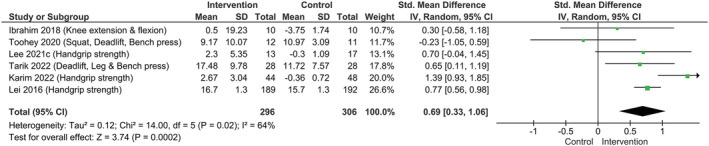
Effect of probiotic supplementation on global muscle strength.

A significant increase in global muscle strength was displayed following probiotic supplementation compared with placebo with a moderate degree of heterogeneity among RCTs (*k* = 6; SMD: 0.69, 95% CI: 0.33–1.06, I^2^ = 64%, *P* = 0.0002) (*Figure* 4).

Based on subgroup analyses, individuals 50 years and above showed a significant improvement in global muscle strength (*k* = 3, SMD: 0.96, 95% CI: 0.52–1.40, I^2^ = 67%, *P* < 0.0001), although this was not observed in individuals below 50 years old (*k* = 3, SMD: 0.31, 95% CI: −0.21–0.84, I^2^ = 35%, *P* = 0.24) (*Figure* S15). Furthermore, longer‐term duration (≥ 12 weeks) elicited a significantly positive changes following probiotic supplementation (*k* = 4, SMD: 0.87, 95% CI: 0.47–1.27, I^2^ = 60%, P < 0.0001) compared with shorter‐term supplementation (*k* = 2, SMD: 0.27, 95% CI: −0.59–1.12, I^2^ = 67%, *P* = 0.54) (*Figure* S16). Details related to subgroup analyses exploring the impact of probiotic supplementation on global muscle strength are shown in *Table* S4.

Sensitivity analysis based on low to moderate risk of bias revealed identical findings with our main analysis (*k* = 5, SMD: 0.83, 95% CI: 0.52–1.14, I^2^ = 50%, *P* < 0.0001) (*Figure* S17).

### Risk of bias of included studies

Seven studies were classified as having some concerns according to RoB2^38^, ^39^, ^40^, ^43^, ^44^, ^48^, ^59^ as displayed in *Figure* S18. Specifically, 10 studies had some concerns due to not providing information regarding treatment allocation.^37^, ^38^, ^39^, ^43^, ^44^, ^45^, ^48^, ^51^, ^53^, ^59^ One study had some concerns given its 1:1 allocation ratio in regard to randomization,^49^ whereas in another study, there was a statistically significant difference between groups.^56^ Additionally, in three studies, the score was high as investigators were aware of the intervention^40^, ^59^ and because no information about allocation treatment, randomization, and table of characteristics was provided.^52^ Thirteen studies had some concerns in which no information regarding staff and participant blinding was detailed,^37^, ^38^, ^39^, ^40^, ^41^, ^43^, ^44^, ^45^, ^46^, ^52^, ^53^, ^57^, ^59^ whereas one study had also some concerns due its single‐blind design.^48^ Furthermore, three studies had some concerns due high participant dropout rates^38^, ^55^, ^58^ and because in two studies no information was provided in relation to participant dropout rate.^39^, ^40^ Finally, one study had some concerns as there was no prespecified plan regarding its outcome measures.^50^

### Publication bias

No publication bias was reported following probiotic supplementation and muscle mass (*z* = −0.7442, *P* = 0.4567) and total lean mass (*z* = −0.1004, *P* = 0.9200). Due to the limited number of studies, publication bias was not performed in relation to global muscle strength (*k* < 10). Funnel plots illustrating publication bias regarding probiotics and muscle mass and total lean mass are shown in *Figure* S19A and S19B, respectively.

## Discussion

In the present study, we systematically reviewed RCTs investigated the effect of probiotics on muscle mass, total lean mass and muscle strength in young and older adults. Our main analyses revealed that probiotic intervention augments both muscle mass that is also supported by subgroup analyses, revealing its effectiveness across individuals of younger age (<50 years), longer duration (≥12 weeks), strain of probiotic supplementation (*Bifidobacterium*) and geographical location (Asia). No such positive effect of probiotics was observed on changes in total lean mass. Interestingly, we found a significant improvement on global muscle strength following probiotic supplementation that was more prominent in older adults (≥50 years), following a longer treatment duration (≥12 weeks).

### Findings from subgroup analyses

Our subgroup analysis based on age revealed a prominent effect of probiotic supplementation on muscle mass in individuals below 50 years of age compared with their older counterparts. Older individuals exhibit a reduced response of muscle protein synthesis (MPS) to anabolic stimuli (i.e. exercise and amino acids) as opposed to younger adults, a phenomenon termed anabolic resistance.^61^, ^62^ This phenomenon is considered a primary factor in the development of sarcopenia. Indeed, it has been reported that older individuals (mean age 71 years) may require almost twice as much protein intake per meal to stimulate MPS to the same degree as younger counterparts (mean age 22 years).^63^ Because habitual dietary protein intakes in older adults are often reported to be inadequate^64^ and protein digestion kinetics (i.e. delayed absorption) also are impaired with advanced age^65^ and given the increasing popularity of plant‐based proteins for environmental and ethical reasons,^66^ the use of probiotics to maximize the quantity of circulating amino acids in order to stimulate MPS and promote skeletal muscle mass is an area that warrants further investigation. Further explanation of our findings may pertain to the chronic elevation in levels of inflammatory biomarkers such as IL‐6, C‐reactive protein (CRP) and TNF‐α, observed in older populations.^67^ Systemic inflammation has been shown to downregulate net muscle protein balance by the suppression of anabolic pathways and upregulation of catabolic pathways [i.e. increased expression of Muscle RING‐finger protein‐1 (MuRF‐1) and atrogin‐1], leading to reductions in muscle mass and quality.^68^ When the diet of aged mice was supplemented with either *Lactobacillus casei* or *Bifidobacterium longum* , probiotics seemed to regulate levels of pro‐inflammatory cytokines such as IL‐6, TNF‐α and monocyte chemoattractant protein‐1 (MCP‐1) and led to enhanced muscle strength and function.^69^ Although the anabolic‐induced responses that could potentially be exerted from probiotic supplementation are mediated, in part, via anti‐catabolic responses suppressing systemic inflammation, it is currently unknown whether this is sufficient to suppress muscle loss in older populations. Our analysis suggests a potential role for the use of probiotic strains to enhance protein digestion and amino acid absorption that may reduce the dose of protein required to stimulate MPS; however, considering anabolic resistance during ageing, their impact on muscle mass is questionable in older populations. Further research is warranted to identify the impact of probiotic supplementation with increased protein intake in older populations and unravel the clinical significance of its anti‐inflammatory properties.

Moreover, we found that longer duration above 12 weeks was superior in inducing muscle mass gains as opposed to short‐term supplementation (<12 weeks), changes that may be explained by the gut microbiota. Several animal^29^, ^70^, ^71^ and human studies^19^, ^20^ have highlighted the importance of the gut microbiome in regulating muscle mass and strength, as well as multiple mechanisms by which it may exert anabolic effects. For example, germ‐free (GF) mice exhibit markedly lower levels of muscle mass/strength/locomotive capability and fatigue resistance when compared with pathogen‐free (PF) mice and abundant gut microbiota.^70^ More importantly, transplantation of the gut microbiota from the PF to the GF mice resulted in increases in skeletal muscle mass and improvements in muscle function of the GF mice. Furthermore, supplementation with probiotics such as *Bacteroides fragilis* ^72^ or co‐housing with PF mice led to improved muscle mass and function in GF mice, further highlighting the importance of the microbiome in the regulation of muscle health. It is worth noting that, in two studies that supplemented probiotic strains in parallel with resistance exercise, the authors did not report significant changes in muscle mass.^39^, ^59^ However, both studies lasted in total for approximately 1 month, which may not be sufficient to exert significant muscle growth.^73^ Future studies incorporating a long‐term resistance exercise with probiotic supplementation would shed light on whether bacterial strains enhance the hypertrophic‐induced responses derived by resistance exercise.

Our subgroup analysis focusing on distinct microbial species depicted a prominent role of *Bifidobacterium* in mediating muscle mass as opposed to supplementation with *Lactobacillus* strains. Both studies incorporated *Bifidobacterium breve* B‐3 compared with placebo for 12 weeks in overweight individuals above 50 years of age.^42^, ^43^ Interestingly, we did not find a beneficial effect on muscle mass after categorization based on older age, and overweight or obese health status, implying that the positive outcomes exhibited by this bacterial strain may be attributed to study duration and any potential skeletal muscle‐specific properties. However, given the small number of studies following *Bifidobacterium* supplementation (*k* = 2) along with the inability to perform subgroup analyses based on different bacterial taxa, we could not establish a causative relationship between probiotic supplementation and increased muscle mass due to *B. breve* B‐3.

Subgroup analysis based on geographical location suggested that a link between muscle mass and probiotic supplementation may be more pronounced in people living in Asia. Recently, a large‐scale survey found a positive association of appendicular lean mass to body weight ratio and *Blautia*, *Bifidobacterium* and *Eisenbergiella* in Japanese adults aged 50.8 (12.8) years.^74^ In our meta‐analysis, both studies in which *Bifidobacterium* supplementation established a beneficial effect on muscle mass were conducted in Japan in adults of similar ages,^42^, ^43^ suggesting a population‐specific association between muscle mass and bacterial strains. No positive findings were found in European countries following probiotic supplementation; however, these results should be treated with caution due to the low number of studies (*k* = 2) and the moderate heterogeneity among studies conducted in Asia (56%).

Finally, we observed a beneficial effect of probiotic supplementation on global muscle strength in individuals 50 years of age and above and following longer‐term protocols (≥12 weeks). Although it is currently unknown why these differences were observed in older versus younger populations, Ni *et al*. found that *L. casei* LC122 and *B. longum* BL986 supplementation for 12 weeks in aged mice promoted significant increase in forelimb grip strength, suggesting a link between muscle strength and the gut microbiota.^69^ Similarly, aged SAMP8 mice supplemented with *Lactobacillus paracasei* PS23 for 12 weeks displayed attenuated age‐related decreases in grip force, exhibiting higher mitochondrial function compared with controls.^26^ Interestingly, these findings are inconsistent with the aforementioned results of probiotics improving muscle mass in younger versus older populations. Whether this is based on the low number of studies related to global muscle strength or the differences among surrogate measures of muscle strength requires further investigation.

### Probiotic mechanisms enhancing muscle function

A number of mechanisms have been proposed to underpin the beneficial role of the microbiome/probiotics in the regulation of muscle mass and function. From a nutritional perspective, the acute post‐prandial elevation of leucine and the presence of other essential amino acids are key determinants of intramyocellular anabolic signalling to trigger MPS, an effect that is potentiated following acute resistance exercise.^75^, ^76^ A possible mechanism by which probiotics may stimulate increases in muscle mass or strength is by improved digestion protein and absorption of constituent amino acids into the bloodstream. Indeed, in vitro studies have revealed that the probiotic strain *Bacillus coagulans* GBI‐30, 6086 (BC30) increases protein digestion and amino acid uptake in a model of the upper gastrointestinal tract.^19^ To translate such effects to human models, Stecker *et al*. administered young men and women a milk protein concentrate, with or without BC30 (1 × 109 CFU) for 2 weeks in a crossover design.^19^ On the final day of supplementation, blood concentrations of amino acids were measured. Findings revealed that the addition of BC30 resulted in a greater postprandial area under the curve (AUC) for arginine and isoleucine, higher peak concentrations of a number of other amino acids (arginine, serine, ornithine, methionine, glutamic acid, phenylalanine, isoleucine, tyrosine, EAAs and total amino acids) and a more rapid rise to peak concentrations for some amino acids (glutamine, citrulline, threonine and alanine). Interestingly, Jäger *et al*. observed equivocal effects using pea protein isolate with or without two strains of *L. paracasei* (5 billion CFU each).^20^ The addition of the probiotics led to significantly increased maximum blood concentrations and AUC of methionine, histidine, valine, leucine, isoleucine, tyrosine, total branched chain amino acids (BCAA) and total EAAs.

Furthermore, the production of short‐chain fatty acids (SCFAs) is a known function of numerous bacterial species within the microbiome, and these compounds have a number of physiological effects on the host.^77^ Mouse models revealed that treatment of GF mice (with reduced muscle mass and function compared with PF controls) with gut microbiota transplants or with SCFAs alone was enough to partially reverse the skeletal muscle impairments observed in the GF animals.^70^ Human observational studies also demonstrate that older adult men with greater levels of lean mass and physical function exhibit higher levels of butyrate‐producing bacteria as well as higher gene counts for butyrate‐producing genes compared with men with lower lean mass.^71^, ^78^ Higher fibre intakes also were associated with a greater butyrate production, which is likely related to the role of dietary fibre as a prebiotic food source for probiotic bacteria.^79^

In addition, creatine degradation has also been observed to be greater in the microbiomes of older compared with younger mice.^80^ Creatine is a non‐protein amino acid found in muscle tissue, among other body compartments where it plays a key role in energy metabolism and muscle contraction.^81^ Muscle creatine levels have been observed to be lower in older populations,^82^ and this decrease may contribute to the multifactorial development of sarcopenia. Taken together, these data support the notion that the gut microbiota/probiotics may play a clinically relevant and mechanistically feasible role in muscle health.

### Strengths and limitations

A strength of this study is the inclusion of meta‐analyses of both total lean mass and muscle mass. We propose that increasing skeletal muscle mass, rather than total lean mass, is a more impactful goal when attempting to improve muscle strength and function or reduce the prevalence of sarcopenia in older adults. Indeed, the European Working Group on Sarcopenia in Older People (EWGSOP) currently uses appendicular skeletal muscle mass as part of the diagnostic criteria for sarcopenia.^1^ Due to the importance of muscle function in addition to muscle mass in ageing populations, it should also be noted that muscle mass (appendicular lean mass index) has been shown to correlate well with various measures of strength such as leg press, knee extension and knee flexion 1RM.^83^ In contrast, although the umbrella term ‘total lean mass’ includes muscle mass, it also includes a great deal of non‐muscle tissue including bone and organs, which may not decline to the extent of muscle mass that is observed in sarcopenia.

Furthermore, although a significant improvement in muscle mass was observed following probiotic supplementation, our results did not detect a notable change in total lean mass by DXA as opposed to BIA, which is thought to be a less accurate and precise tool for the measurement of body composition. This circumstance may raise some questions as to the true clinical relevance of the improvements found in muscle mass after probiotic supplementation, as DXA is considered to be a reliable clinical tool for diagnosing and monitoring the age‐related loss of muscle mass. Finally, the duration of interventions was not superior to 12 weeks in all the considered studies, and as such, the long‐term effects of probiotic supplementation on muscle mass and strength remain unknown. To be clinically useful, any potential treatment against sarcopenia should prove effective in the long term, as sarcopenia represents a chronic phenomenon affecting health outcomes and quality of life of older subjects beyond the horizon of just few weeks of treatment.

## Conclusions

In conclusion, this is the first systematic review and meta‐analysis to demonstrate that probiotic interventions augment global muscle strength and muscle mass, although no such positive effects were observed for total lean mass. Pertaining to muscle mass, the results of our meta‐analysis highlight the positive effect of probiotic supplementation across populations below the mean age of 50, utilizing a longer‐term (≥12 weeks) treatment duration, and specifically consuming *Bifidobacterium*, in Asian countries. This field warrants further research to elucidate the mechanisms of action in which probiotics may induce anabolic responses and optimize strategies related to the use of appropriate probiotic strains for the augmentation of both muscle mass and strength in a wide range of population groups.

## Funding

This study received no external funding.

## Conflict of interest

The authors declare no conflicts of interest.

## Supporting information


**Figure S1.** Effect of probiotic supplementation on muscle mass in healthy adults and adults with overweight or obesity.

**Figure S2.** Effect of probiotic supplementation on muscle mass in adults below 50 years of age or 50 years and above.

**Figure S3.** Effect of probiotic supplementation on muscle mass during short‐term (< 12 weeks) and long‐term (≥ 12 weeks) duration.

**Figure S4.** Effect of probiotic supplementation on muscle mass utilizing different bacterial species.

**Figure S5.** Effect of probiotic supplementation on muscle mass based on geographical location.

**Figure S6.** Effect of probiotic supplementation on muscle mass according to body composition assessment tool.

**Figure S7.** Effect of probiotic supplementation on muscle mass based on risk of bias.

**Figure S8.** Effect of probiotic supplementation on lean body mass in healthy adults, adults with overweight or obesity and athletic individuals.

**Figure S9.** Effect of probiotic supplementation on lean body mass in adults below 50 years of age or 50 years and above.

**Figure S10.** Effect of probiotic supplementation on lean body mass during short‐term (< 12 weeks) and long‐term (≥ 12 weeks) duration.

**Figure S11.** Effect of probiotic supplementation on lean body mass utilizing different bacterial species.

**Figure S12.** Effect of probiotic supplementation on lean body mass based on geographical location.

**Figure S13.** Effect of probiotic supplementation on lean body mass according to body composition assessment tool.

**Figure S14.** Effect of probiotic supplementation on lean body mass based on risk of bias

**Figure S15.** Effect of probiotic supplementation on global muscle strength in adults below 50 years of age or 50 years and above.

**Figure S16.** Effect of probiotic supplementation on global muscle strength during short‐term (< 12 weeks) and long‐term (≥ 12 weeks) duration.

**Figure S17.** Effect of probiotic supplementation on global muscle strength based on risk of bias.

**Figure S18.** Quality assessment of the included studies based on the Cochrane risk‐of‐bias tool for randomised trials (RoB 2).

**Figure S19.** **(A and B)** Funnel plot of muscle mass (kg) and lean body mass (kg) used to illustrate publication bias. Inverse of the standard error has been plotted on the y axis with the standardized mean difference plotted on the x axis. Each circle within the plot represents an individual study, while the circles outside the confidence region have been identified as studies with potential risk of publication bias.

**Table S1.** Search terms employed in the screening based on title, abstract and keywords in the literature search.

**Table S2.** Subgroup analyses for studies evaluating the impact of probiotic supplementation on muscle mass.
**Table S3.** Subgroup analyses for studies evaluating the impact of probiotic supplementation on total lean mass.
**Table S4.** Subgroup analyses for studies evaluating the impact of probiotic supplementation on global muscle strength.
