# Supplementary material for: Impact of probiotics on muscle mass, muscle strength and lean mass: a systematic review and meta‐analysis of randomized controlled trials
Source: J Cachexia Sarcopenia Muscle. 2022 Nov 22;14(1):30–44. doi: 10.1002/jcsm.13132 (PMC9891957; doi:10.1002/jcsm.13132)

**FigureS18.** Quality assessment of the included studies based on the Cochrane risk-of-bias tool for randomised trials (RoB 2).


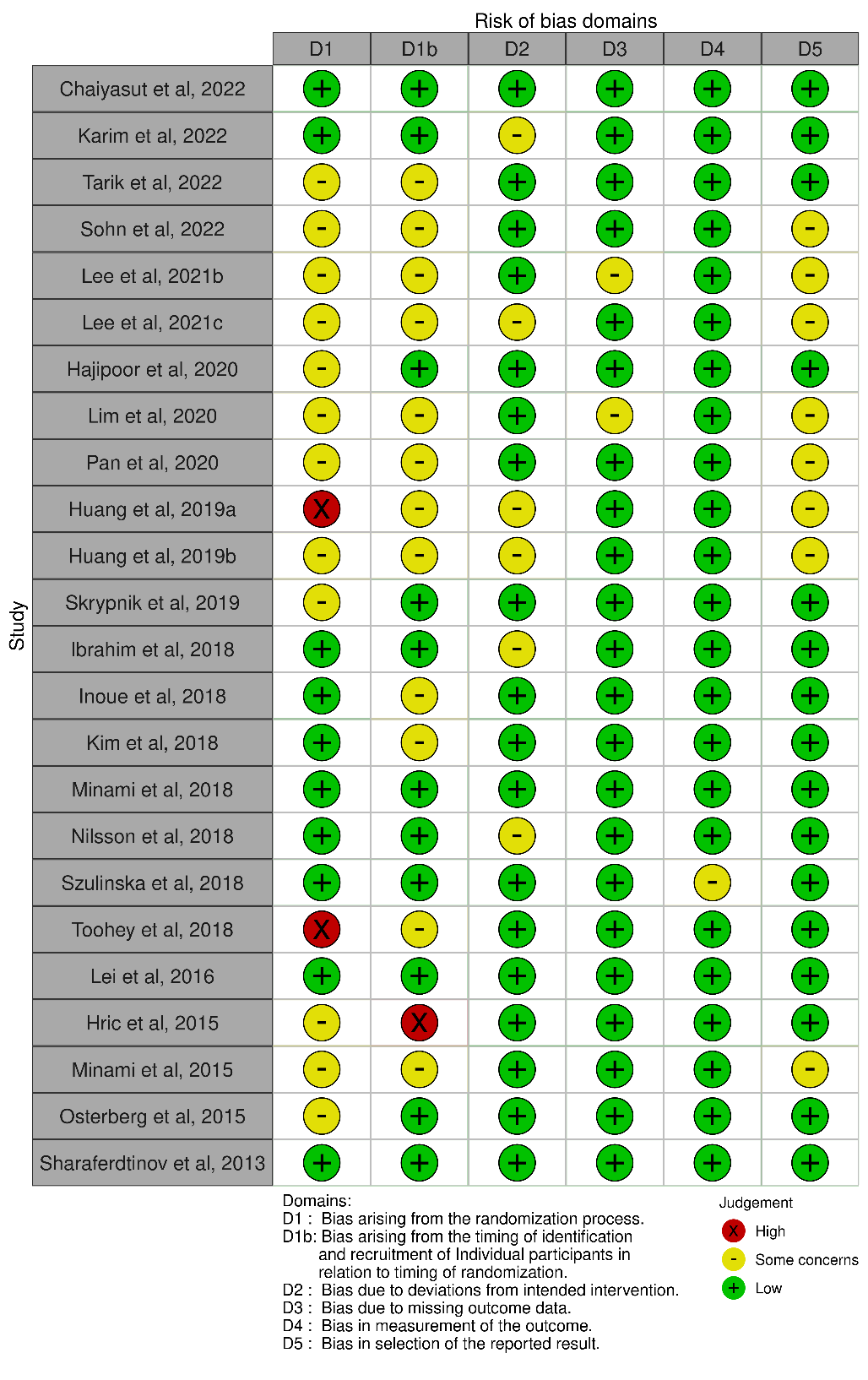

Supplement: Supplementary file 18 — Figure S18. Quality assessment of the included studies based on the Cochrane risk‐of‐bias tool for randomised trials (RoB 2). [file JCSM-14-30-s013.docx]
