# Supplementary material for: Impact of probiotics on muscle mass, muscle strength and lean mass: a systematic review and meta‐analysis of randomized controlled trials
Source: J Cachexia Sarcopenia Muscle. 2022 Nov 22;14(1):30–44. doi: 10.1002/jcsm.13132 (PMC9891957; doi:10.1002/jcsm.13132)
Supplement: Supplementary file 20 — Table S1. Search terms employed in the screening based on title, abstract and keywords in the literature search. [file JCSM-14-30-s001.docx]

**Table S1.**Search terms employed in the screening based on title, abstract and keywords in the literature search.

| **Database** | **Search terms** |
| --- | --- |
|  |  |
| PubMed  Cochrane Library  Web of Science  Scopus | (humans OR adults OR men OR women OR males OR females) AND (probiotic* OR lactobacill* OR bifidobacteri* OR Saccharomyces OR Escherichia coli OR kefir) AND (“skeletal muscle” OR “muscle mass” OR “muscle strength” OR “handgrip strength” OR “appendicular muscle mass” OR “appendicular lean mass” OR “lean body mass” OR “lean mass” OR “fat free mass” OR “knee extension” OR “knee flexion” OR “quadriceps strength” OR “lower limb strength”)  (humans OR adults OR men OR women OR males OR females) AND (probiotic* OR lactobacill* OR bifidobacteri* OR Saccharomyces OR Escherichia coli OR kefir) AND (“skeletal muscle” OR “muscle mass” OR “muscle strength” OR “handgrip strength” OR “appendicular muscle mass” OR “appendicular lean mass” OR “lean body mass” OR “lean mass” OR “fat free mass” OR “knee extension” OR “knee flexion” OR “quadriceps strength” OR “lower limb strength”)  TS=(humans OR adults OR men OR women OR males OR females) AND (probiotic* OR lactobacill* OR bifidobacteri* OR Saccharomyces OR Escherichia coli OR kefir) AND (“skeletal muscle” OR “muscle mass” OR “muscle strength” OR “handgrip strength” OR “appendicular muscle mass” OR “appendicular lean mass” OR “lean body mass” OR “lean mass” OR “fat free mass” OR “knee extension” OR “knee flexion” OR “quadriceps strength” OR “lower limb strength”)  TITLE-ABS-KEY=(humans OR adults OR men OR women OR males OR females) AND (probiotic* OR lactobacill* OR bifidobacteri* OR Saccharomyces OR Escherichia coli OR kefir) AND (“skeletal muscle” OR “muscle mass” OR “muscle strength” OR “handgrip strength” OR “appendicular muscle mass” OR “appendicular lean mass” OR “lean body mass” OR “lean mass” OR “fat free mass” OR “knee extension” OR “knee flexion” OR “quadriceps strength” OR “lower limb strength”) |
