# Supplementary material for: Impact of probiotics on muscle mass, muscle strength and lean mass: a systematic review and meta‐analysis of randomized controlled trials
Source: J Cachexia Sarcopenia Muscle. 2022 Nov 22;14(1):30–44. doi: 10.1002/jcsm.13132 (PMC9891957; doi:10.1002/jcsm.13132)
Supplement: Supplementary file 21 — Table S2. Subgroup analyses for studies evaluating the impact of probiotic supplementation on muscle mass. Table S3. Subgroup analyses for studies evaluating the impact of probiotic supplementation on total lean mass. Table S4. Subgroup analyses for studies evaluating the impact of probiotic supplementation on global muscle strength. [file JCSM-14-30-s018.docx]

**Table S2.** Subgroup analyses for studies evaluating the impact of probiotic supplementation on muscle mass.

|  | Subgroup | N^o^ of studies | SMD (95% CI) | P-value | I^2^ |
| --- | --- | --- | --- | --- | --- |
| Age | < 50 years  ≥ 50 years | *k* = 4  *k* = 6 | 0.47 (0.03, 0.91)  0.41 (-0.06, 0.88) | P = 0.04  P = 0.09 | 35%  69% |
| Duration | < 12 weeks  ≥ 12 weeks | *k* = 6  *k* = 4 | 0.42 (-0.23, 1.07)  0.39 (0.15, 0.63) | P = 0.20  P = 0.002 | 73%  0% |
| Health  status | Overweight/  Obese  Healthy untrained | *k* = 5  *k* = 4 | 0.46 (-0.06, 0.97)  0.43 (-0.20, 1.06) | P = 0.08  P = 0.18 | 66%  63% |
| Species | Lactobacillus  Bifidobacterium | *k* = 7  *k* = 2 | 0.48 (-0.05, 1.01)  0.37 (0.02, 0.73) | P = 0.08  P = 0.04 | 69%  0% |
| Geographical location | Europe  Asia | *k* = 2  *k* = 7 | -0.15 (-0.69, 0.40)  0.61 (0.22, 1.01) | P = 0.60  P = 0.002 | 0%  56% |

SMD, standardised mean difference

**Table S3.** Subgroup analyses for studies evaluating the impact of probiotic supplementation on total lean mass.

|  | Subgroup | N^o^ of studies | SMD (95% CI) | P-value | I^2^ |
| --- | --- | --- | --- | --- | --- |
| Age | < 50 years  ≥ 50 years | *k* = 5  *k* = 7 | -0.03 (-0.26, 0.19)  -0.03 (-0.25, 0.19) | P = 0.79  P = 0.62 | 0%  0% |
| Duration | < 12 weeks  ≥ 12 weeks | *k* = 4  *k* = 8 | -0.01 (-0.32, 0.30)  -0.04 (-0.23, 0.14) | P = 0.96  P = 0.66 | 0%  0% |
| Health  status | Overweight/Obese  Healthy untrained  Athletic | *k* = 8  *k* = 2  *k* = 2 | -0.03 (-0.21, 0.15)  -0.05 (-0.57, 0.46)  -0.02 (-0.46, 0.42) | P = 0.73  P = 0.84  P = 0.92 | 0%  0%  0% |
| Species | Lactobacillus  Bifidobacterium  Lactobacillus & Bifidobacterium & Lactococcus | *k* = 7  *k* = 3  *k* = 2 | -0.00 (-0.20, 0.20)  -0.03 (-0.40, 0.33)  -0.16 (-0.57, 0.24) | P = 0.99  P = 0.85  P = 0.44 | 0%  0%  0% |
| Geographical location | Europe  Asia | *k* = 3  *k* = 6 | -0.04 (-0.35, 0.27)  -0.03 (-0.24, 0.19) | P = 0.82  P = 0.80 | 0%  0% |
| Muscle mass assessment tool | BIA  DXA | *k* = 4  *k* = 8 | -0.14 (-0.46, 0.17)  0.01 (-0.18, 0.19) | P = 0.37  P = 0.95 | 0%  0% |

SMD, standardised mean difference

**Table S4.** Subgroup analyses for studies evaluating the impact of probiotic supplementation on global muscle strength.

|  | Subgroup | N^o^ of studies | SMD (95% CI) | P-value | I^2^ |
| --- | --- | --- | --- | --- | --- |
| Age | < 50 years  ≥ 50 years | *k* = 3  *k* = 3 | 0.23 (-0.03, 0.50)  0.96 (0.52, 1.40) | P = 0.09  P < 0.001 | 18%  67% |
| Duration | < 12 weeks  ≥ 12 weeks | *k* = 2  *k* = 4 | 0.20 (-0.13, 0.54)  0.78 (0.39, 1.17) | P = 0.24  P < 0.001 | 39%  59% |

SMD, standardised mean difference
